# Mitogenome Variations in a Global Population of *Aspergillus fumigatus*

**DOI:** 10.3390/jof9100995

**Published:** 2023-10-08

**Authors:** Veronica Thorn, Jianping Xu

**Affiliations:** Department of Biology, Institute of Infectious Diseases Research, McMaster University, Hamilton, ON L8S 4K1, Canada; thornv2@mcmaster.ca

**Keywords:** human pathogen, *Aspergillus*, mitochondria, single nucleotide polymorphism, clonal expansion, recombination, copy number variation

## Abstract

*Aspergillus fumigatus* is a ubiquitous, critical priority human fungal pathogen. Despite its clinical importance, there is limited knowledge regarding the variations of the genome within mitochondria, the powerhouse organelle within eukaryotic cells. In this study, we leveraged publicly available, raw, whole genome sequence data isolates from 1939 to investigate the variations in the mitochondrial genomes of *A. fumigatus*. These isolates were isolated from 22 countries on six continents, as well as from outer space and from within the International Space Station. In total, our analysis revealed 39 mitochondrial single nucleotide polymorphisms (mtSNPs) within this global sample, and, together, these 39 mtSNPs grouped the 1939 isolates into 79 mitochondrial multilocus genotypes (MLGs). Among the 79 MLGs, 39 were each distributed in at least two countries and 30 were each shared by at least two continents. The two most frequent MLGs were also broadly distributed: MLG11 represented 420 isolates from 11 countries and four continents and while MLG79 represented 418 isolates from 18 countries and five continents, consistent with long-distance dispersals of mitogenomes. Our population genetic analyses of the mtSNPs revealed limited differentiation among continental populations, but highly variable genetic differences among national populations, largely due to localized clonal expansions of different MLGs. Phylogenetic analysis and Discriminant Analysis of Principal Components of mtSNPs suggested the presence of at least three mitogenome clusters. Linkage disequilibrium, Index of Association, and phylogenetic incompatibility analyses collectively suggested evidence for mitogenome recombination in natural populations of *A. fumigatus*. In addition, sequence read depth analyses revealed an average ratio of ~20 mitogenomes per nuclear genome in this global population, but the ratios varied among strains within and between certain geographic populations. Together, our results suggest evidence for organelle dynamics, genetic differentiation, recombination, and both widespread and localized clonal expansion of the mitogenomes in the global *A. fumigatus* population.

## 1. Introduction

*Aspergillus fumigatus* is a ubiquitous airborne saprophytic fungus, and a leading cause of human fungal infections [1]. Playing an important role in the carbon and nitrogen cycles, *A. fumigatus*’ ecological niche is primarily in organic debris and soil [2]. However, they can grow in a broad range of environmental conditions, including indoor, outdoor, and aqueous, and even extraterrestrial environments such as the International Space Station (ISS) [3,4]. Reproducing mainly asexually, *A. fumigatus* spreads via sporulation of minute conidia, roughly 2–3 µm in diameter, light enough for airborne dissemination and small enough to penetrate lung alveoli [5]. The combination of widespread environmental habitats and airborne spores leads to a high frequency of incidental inhalation every day for the majority of people throughout the world [6].

Despite the near constant inhalation of spores, a healthy host’s innate immune system is usually able to eliminate the foreign cells, such that clinical symptoms are mild and rarely seen [6]. However, in immunocompromised patients, such as transplant recipients, leukemia patients, and those with acquired immunodeficiency syndrome (AIDS), inhaled *A. fumigatus* spores often develop into invasive aspergillosis (IA), with mortality rates reaching as high as 90% [5]. Consequently, the World Health Organization (WHO) has designated *A. fumigatus* a Critical Priority fungal pathogen [7]. Antifungal treatment is essential for effectively clearing the fungal pathogen in acute aspergillosis cases [6]. However, shared fundamental metabolic pathways between fungal and human cells limit the availability of antifungal drug targets and treatment options. Furthermore, the emergence of drug-resistant strains in both environmental and clinical settings has contributed to a noticeable increase in mortality rates, surpassing non-resistant infections by more than 25% [8,9].

For effective growth and reproduction both in vitro and in vivo, like most other eukaryotes, *A. fumigatus* requires energy generated by mitochondria through oxidative respiration. In addition, aside from energy generation, fungal mitochondria have also been implicated in various cellular processes, including temperature and oxidative stress responses, pathogenicity, antifungal resistance, iron homeostasis, and fungal dormancy [10,11,12,13,14,15]. In most fungal cells, there may be multiple mitochondria, with each containing one or more mitochondrial genome (mitogenome) that replicates independently of the nuclear genome and of the cell cycle [16,17]. Typically, each mitogenome is a single circular DNA molecule. The core gene content in fungal mitogenomes is relatively well conserved among species [18]. However, among species, the mitogenome sizes can vary significantly, from about 20 kb to over 200 kb [17,19,20]. Among strains within the same species, variations in mitogenome size and gene content have also been observed [17]. The inheritance of mitochondria in most eukaryotic organisms is uniparental, typically maternal, following a non-Mendelian pattern [16]. However, other modes of inheritance have been observed in fungi (for an in-depth review, see [17]). At present, the mode of mitochondrial inheritance in *A. fumigatus* and potential variations among strains in their mitogenomes of this fungal pathogen are unknown.

Since mitogenomes are small, relatively abundant, and well conserved yet often distinct among species, mitogenome analysis is a popular method for population genetics, physiological, and evolutionary studies [21,22]. Indeed, for most animals the mitochondrial cytochrome c oxidase 1 gene (COI) has been adopted as a DNA barcode, a standard for species identification in many animal taxa [23]. In fungi, however, COI is less commonly used due to the occurrence of introns within the gene and limited sequence divergence among species in certain fungal lineages [24]. Nevertheless, due to their small size and relatively high abundance within each cell, mitogenomic analysis could provide insights into fungal evolution, population structure, and reproduction at a fraction of the cost of conventional nuclear genomic sequencing [19].

In recent years, there has been a notable increase in the number of studies focusing on mitochondrial genomics. However, the application of mitochondria-based analyses to investigate fungal population structure, particularly within the *Aspergillus* species, is not well represented in the literature. Furthermore, investigations into the intraspecies structure or whole-genome level analysis of mitogenomic single nucleotide polymorphisms (mtSNPs) in *A. fumigatus* are currently nonexistent. Previous studies examining interspecies population dynamics and phylogenetics in the *Aspergillus* genus have predominantly relied on a single gene, such as cytochrome b or concatenated alignments of multiple mitochondrial genes, most of which were conducted more than a decade ago. Notably, Kozlowski and Stepien [25] pioneered the use of mitochondrial restriction enzyme analysis to establish interspecies phylogenetic relationships within the *Aspergillus* genus. Subsequent studies by Wang et al. [26,27] successfully investigated the phylogenetic relationships between *A. fumigatus* and other pathogenic *Aspergillus* species using cytochrome b gene sequences. More recently, Joardar et al. [19] published the first complete mitogenomes of nine *Aspergillus* and *Penicillium* species, including employing 14 concatenated mitochondrial proteins for inter-species and inter-genus phylogenetic analyses. Although relatively less common than single gene focused assays, genome-wide mtSNPs for phylogenetic and population analyses has been successfully demonstrated in studies on crops, cattle, humans, and selected fungi [22,28,29,30,31].

With the increasing abundance and availability of whole genome sequencing (WGS) reads in *A. fumigatus*, we conducted a large-scale intraspecific analysis of mtSNPs in a global *A. fumigatus* population. Specifically, raw sequencing reads of about 2000 isolates of *A. fumigatus* were available (as of 2 May 2023) in the National Centre for Biology Information’s (NCBI) Sequence Read Archive (SRA), each including both the nuclear and mitochondrial sequence reads. While the nuclear genomes of these strains have been analyzed and published in a diversity of papers and journals [32,33,34,35,36], the accompanying mitochondrial DNA (mtDNA) sequences have received limited attention. Furthermore, due to the higher abundance of mtDNA molecules than nuclear chromosomal DNA in the cell and the nature of gene sequencing, the sequence read depths of mtDNA are generally high, enabling accurate SNP calls [19]. Our study aimed to identify mtDNA sequence variation, examine the potential geographic patterns of the observed variation, and investigate evidence of recombination in shaping mitogenome diversity in this clinically significant human fungal pathogen.

## 2. Materials and Methods

### 2.1. Data Acquisition and Processing

NCBI’s SRA, which includes raw data from published experiments, the European Bioinformatics Institute (EBI), and the DNA Database of Japan (DDBJ), served as the data source and repository for this study. A query was performed on 2 May 2023 on the SRA to identify all accessions associated with “*Aspergillus fumigatus*” and Whole Genome Sequencing (WGS) and Whole Genome Amplicon (WGA) assay types. The raw sequence data for each accession was obtained using the sra-toolkit (v3.0.0), and subsequently subjected to quality checking and trimming using Fastp (v0.23.1) [37].

To ensure data integrity, reads were filtered based on quality (requiring at least 60% of bases with a phred score of 30 or higher), length (minimum of 30 base pairs), and complexity. The filtered reads were aligned to a reference *A. fumigatus* mitochondrial genome (strain Af293, GenBank JQ346808.1) using Burrows–Wheeler Aligner (BWA-MEM v0.7.17-r1188) [38]. 

The alignments were further processed using samtools (v1.17) [39] to ensure quality, length, and alignment depth. Strains with alignments with an average sequence depth below 10× were considered poor quality and excluded from further analysis. Duplicate reads were removed using MarkDuplicates in the Picard tool (v2.26.3) and genetic variants were called using GATK tools, including HaplotypeCaller (with the ‘-ERC GVCF’ flag), GenomeDBImport, and GenotypeGVCFs (v4.2.5.0) [40]. The resultant multisample VCF file was subjected to additional filtering using bcftools (1v.16) [39] to remove insertions/deletions.

Country and continent of origin for each strain was initially determined from associated metadata collected from NCBI. In cases where metadata was incomplete, a manual literature search was conducted to confirm the origin information. Accessions with unconfirmed or artificial origins were labelled as having “Unknown” geographic origins. All isolates retrieved and analyzed, including their relevant metadata, and are shown in Supplementary Table S1.

### 2.2. Data Analysis

#### 2.2.1. Phylogeny

To investigate the relationships among strains and mitogenomes, we conduct phylogenetic analysis using concatenated SNPs. The SNPs were aligned using the program vcf2phylip (v2.0) [41], and the alignment was used to construct a neighbor joining phylogenetic tree using the R programming language (v4.2.3) of the fastreeR package (v1.2.0) [42]. The tree was rooted using the ape package (v5.7) [43] with an *A. fischeri* isolate as the outgroup. The Interactive Tree Of Life (iTOL) web viewer (v5) [44] was used to visualize the resulting tree and incorporate sample metadata.

#### 2.2.2. Population Genetic Analysis

To investigate the potential influence of geographic isolation on mitogenomic population structure, population genetic analyses were performed using the R statistical software and the R package poppr (v.2.9.4) [45]. The first step involved calculating basic diversity statistics and examining the distribution of unique multi-locus genotypes (MLGs) based on mitogenome SNPs present in the entire population. From the initial multisample VCF, four distinct datasets were created to facilitate subsequent analyses. These datasets included: (1) the full dataset without the *A. fischeri* root, (2) a clone-corrected (CC) full pooled dataset, (3) a dataset excluding countries with fewer than nine samples and samples with unknown or non-geographic origins, and (4) a country wise CC dataset where only one representative for each unique MLG type from each country was included.

Population structure was explored by performing a Discriminant Analysis of Principal Components (DAPC) on both the full and country wise clone corrected datasets. For both datasets, Kernel (K) values were chosen from where the Bayesian Information Criterion (BIC) began to decrease minimally, based on 10 repetitions using the find.clusters function from the adegenet R package (v2.1.10) [46]. DAPC was then applied to the dataset clustered using the chosen K values using the dapc function from the adegenet R package [46]. For the full dataset, a K of three was chosen and both three and four were chosen for the country-wise clone-corrected dataset for comparison.

The impact of geography on genetic distribution was further explored through an Analysis of MOlecular VAriance (AMOVA). The country-filtered and country-wise CC datasets were subjected to AMOVA at both the country and continental levels. Additionally, pairwise Nei’s Fst values were calculated between pairs of countries to assess the genetic differentiation among them. To evaluate the correlation between geographic and genetic distances, a Mantel test was performed using the non-CC filtered dataset. This analysis involved comparing the geographic distances (as measured by latitude and longitude) with the corresponding genetic distances (measured as pairwise Nei’s Fst in this implementation) between countries. Significance testing was conducted using 999 permutation bootstrap iterations, with the null hypothesis assuming no genetic differentiation among the geographic populations.

#### 2.2.3. Recombination and Linkage Disequilibrium

Three tests were conducted to assess the presence of recombination in the mitogenome of *A. fumigatus*. The first test employed the common multilocus Index of Association (I_A_) [47,48] to investigate allele associations across different loci. To determine the statistical significance of the observed I_A_, we generated 999 artificially recombined datasets through random allele permutation while maintaining allelic proportions at each locus, and evaluated each for I_A_. A significantly higher observed I_A_ compared to the randomly recombined permutations would indicate a lack of random recombination in the dataset, leading to the rejection of the null hypothesis of panmixia (i.e., no association of alleles between different loci). To facilitate comparisons between studies, we standardized I_A_ by dividing it by the number of loci analyzed, yielding the measure rD. I_A_ and rD calculations were performed on the clone-corrected dataset, where only one representative of each unique MLG was included. These calculations were conducted using the R package poppr [45].

To further investigate the potential recombination patterns in the mitogenome, the second test investigated allelic associations between all pairs of SNP loci across the mitogenome, using the clone-corrected pooled dataset. For each loci pair, both the coefficient (D) of linkage disequilibrium (LD) and the correlation coefficient (r^2^) were computed, with statistical significance assessed through a χ^2^ test against the null hypothesis of panmixia (i.e., no association of alleles between different loci). Considering the extensive number of locus pairs, *p*-values from the pairwise χ^2^ tests were adjusted using the Bonforroni method. Both LD calculations and χ^2^ analyses were performed in R.

The third test complimented the LD analysis as it focused on determining the proportion of phylogenetically compatible pairs of loci using the Four Gamete Test between pairs of SNP loci. Phylogenetic compatibility of two loci is established when it is possible to account for all observed genotypes in the sample without inferring homoplasy (reversals, parallelisms, or convergences) or recombination [22]. For biallelic SNP loci observed in this study, a maximum of four possible haploid genotypes per pair of loci can exist. If no more than three of the four genotypes are observed in the sample, all combinations can be explained without homoplasy or recombination, indicating phylogenetic compatibility. However, when all four possible genotypes are present, the presence of all four cannot be explained without invoking homoplasy or recombination, signifying phylogenetic incompatibility [22]. The analysis was performed in R, and bootstrap procedures were manually implemented with the assistance of the shufflepop function in the poppr package [45]. Visualizations for both LD and the Four Gamete test were generated using the corrplot R package [49].

#### 2.2.4. Mitochondria to Nuclear Genome Comparison

To assess the relative abundance of the mitogenome and the nuclear genome within a cell, we used two methods. In the first, we obtained the average read depth of a single-copy gene, *cyp51A* (GeneID: 3509526), to represent the nuclear genome and compared it with the average read depth of the mitogenome. The *cyp51A* read depth for each strain was obtained by aligning the adapter-trimmed and quality-filtered reads from each sample to the reference genome Af293 using the same methodology as described earlier. The entire mitogenome sequence was used to obtain the average read depth of the mitogenome for each strain. The read depth for each nucleotide position for both the *cyp51A* gene and the mitogenome in each strain was determined using samtools depth (v1.17) [39], incorporating the unmapped positions through the inclusion of the ‘-a’ flag. This approach ensured that even the unmapped positions were accounted for in the read depth calculation for the specific gene fragments. The resulting read depths were then averaged across all nucleotide positions, allowing for a more accurate estimation of the average depth by considering both mapped and unmapped sites. For each sample, we calculated the mitogenome to nuclear genome ratio by dividing the average mitogenome read depth by the *cyp51A* read depth.

In the second method, we estimated the nuclear genome read depth based on whole-genome sequencing coverage. To estimate coverage, the total count of bases in each sequence file that remained post quality filtering and trimming was divided by the aggregate genome size of the *A. fumigatus* reference strain, Af293 (GenBank: GCA_000002655.1), minus that of the mitogenome. The obtained coverage was then compared with the mitogenome coverage described in method #1 above. The obtained ratios were then analyzed for their potential phylogenetic and geographic patterns.

## 3. Results

In this study, we retrieved the raw DNA sequence reads of all the strains of *A. fumigatus* that had their whole-genome sequences deposited in the SRA by 2 May 2023, and one *Aspergillus fischeri* sample used as an outgroup for later phylogenetic analysis. The retrieved 1939 strains came from 22 countries representing six continents, as well as from outer space (2 isolates) and from within the International Space Station (2 isolates) (Table 1). At the continental level, 53.43% of the isolates were from Europe, followed by 29.55% from North America, 9.33% from Asia, 1.24% from Oceania, 0.46% from Africa, and 0.1% from South America. About 5.9% of the isolates did not have any associated geographic information (Table 1). At the country level, USA had the highest number of isolates sequenced, followed by the Netherlands, Germany, United Kingdom, France, Japan, and Ireland, while the remaining 16 countries each contributed less than 30 isolates (Table 1).

### 3.1. Distribution of Multilocus Genotypes Based on Mitogenome SNPs

Using the mitogenome sequence of Af293 as reference and the retrieved sequence reads from the 1939 isolates, we identified a total of 39 SNP sites in this global collection of isolates. Of these 39 SNPs, 15 (38.5%) were intergenic while 24 (61.5%) were in protein-coding regions. Among the 24 SNPs in protein-coding regions, 3 (7.69%) were synonymous and 21 (53.85%) were non-synonymous. All nonsynonymous mutations were predicted to have low-to-moderate effects on protein structure and functions (Supplementary Table S2). These 39 SNPs enabled the identification of 79 mitogenome multilocus genotypes (MLGs). Similar to the sample size pattern, at the continental level, the largest number of MLGs were found in Europe, followed by North America, Asia, Oceania, Africa, and South America. At the country level, the highest number of MLGs were from the USA, followed by Germany, France, the UK, Japan, Ireland, the Netherlands, and Spain. The remaining 14 countries each had less than 10 mitogenome MLGs in the SRA. 

**Figure 1:**
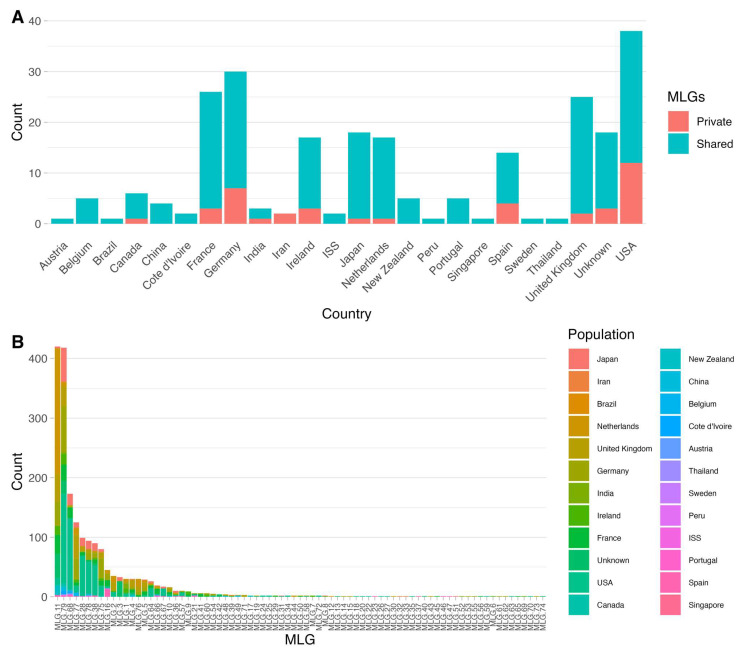
Geographic distributions of mitochondrial multilocus genotypes. (**A**) Stacked bar graph displaying the counts of private and shared MLGs per region at the “country” level for each region included in the total dataset. (**B**) Stacked bar graph displaying the geographic distribution of each of the 79 MLGs. Regions at the “country” level are denoted by color, and the y-axis shows the raw strain count for each MLG in the total dataset.

Among the 79 MLGs, 39 were shared by at least two countries and 30 were shared by at least two continents. The remaining 40 were each found in only one country so far and 35 of these were only represented by one isolate each (Figure 1). The two most frequently shared MLGs, MLG11 and MLG79, were geographically broadly distributed, representing 420 isolates from 11 countries and 418 isolates from 18 countries, respectively (Figure 1). The remaining MLGs were relatively less frequent and showed variable geographic distributions. Among the 77 remaining MLGs, 19 were each shared among at least three countries by 10 or more isolates. Interestingly, MLG79 was the MLG of the reference strain Af293. Between the two most frequent MLGs (i.e., MLG11 and MLG79), they differed from each other at 10 of the 39 SNP sites. Among these 10 sites, 6 were missense variants, which resulted in amino acid substitutions (Supplementary Table S2). All six amino acid substitutions were considered to have moderate effect on protein structure and function. In addition, none of the six nonsynonymous substitutions were unique to MLG11 and other MLGs had variable numbers of these six SNPs (Supplementary Table S2). 

### 3.2. Phylogenetic Relationships among Strains and MLGs Based on Mitogenome SNPs

**Figure 2:**
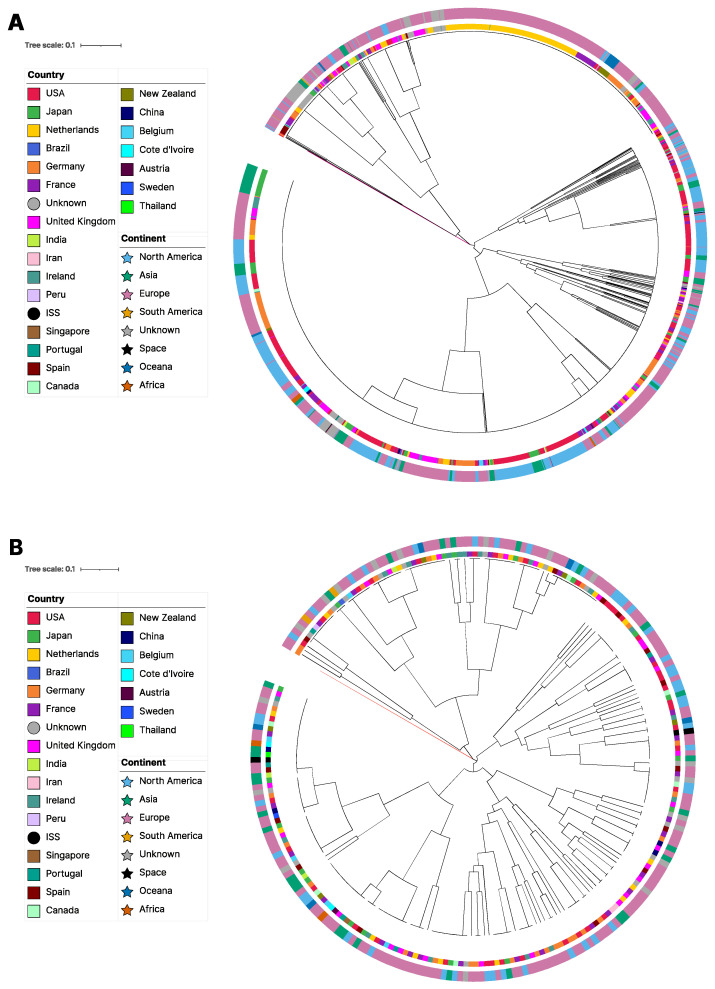
Neighbor joining phylogenetic trees showing relationships among *A. fumigatus* MLGs and strains. (**A**) all 1939 samples were included, with *A. fisherii* serving as outgroup, with its branch highlighted in red. (**B**) Only MLGs after clone correction at the country level are shown (n = 244). In both A and B, the inner ring denotes the country of origin and the outer ring denotes continent of origin. In the legend, countries are denoted by squares and different colors, with circles emphasizing extra-terrestrial or unknown categories. Continents are represented with stars and different colors to further distinguish them from countries in the legend.

The relationships among strains and MLGs based on mitogenome SNPs are shown in Figure 2. This neighbor-joining phylogenetic tree was constructed from the concatenated 39 SNPs. Overall, a few country- and/or continent-based small clusters of strains and MLGs were found (Figure 2). For example, small clusters containing two or more closely related MLGs were found within several countries such as the US, Germany, Japan, Spain, UK, etc. However, the overall phylogeny revealed that all major clusters of MLGs contained strains and MLGs from different countries and/or different continents. Taken together, the results are consistent with frequent gene flow of mitogenomes among geographic regions, but also show that there are unique mitochondrial genotype(s) within many geographic regions.

### 3.3. Genetic Clusters of the Global Mitogenome MLGs

**Figure 3:**
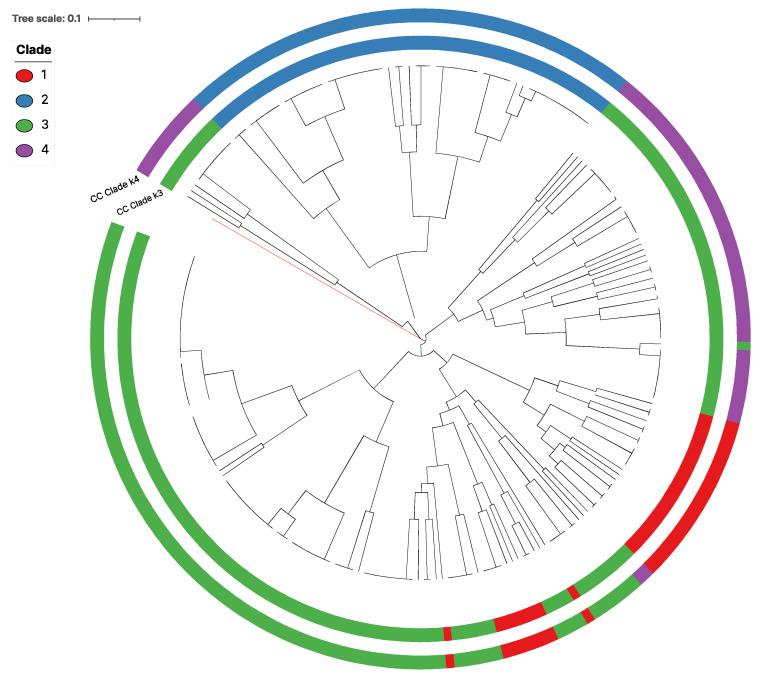
Country wise clone-corrected (CC) phylogenetic tree with DAPC analysis groupings. Branch highlighted in red shows the outgroup taxon *A. fisherii*. Different colors represent different clade assignment. The two rings were generated using two different DAPC parameters. The inner ring was generated using the kernel k = 3. The outer ring was created using the kernel k = 4.

We analyzed whether the 79 MLGs could be grouped into distinct genetic clusters. To accomplish this, we used the Discriminant Analysis of Principal Components (DAPC) of pairwise distances among MLGs. The DAPC analysis showed a clustering pattern very similar to that observed in the cladograms, with three groups appearing as the most parsimonious. However, there were four cases of incongruence between the DAPC grouping of three genetic clusters and the NJ tree, highlighted by different colors shown in Figure 3. 

**Figure 4:**
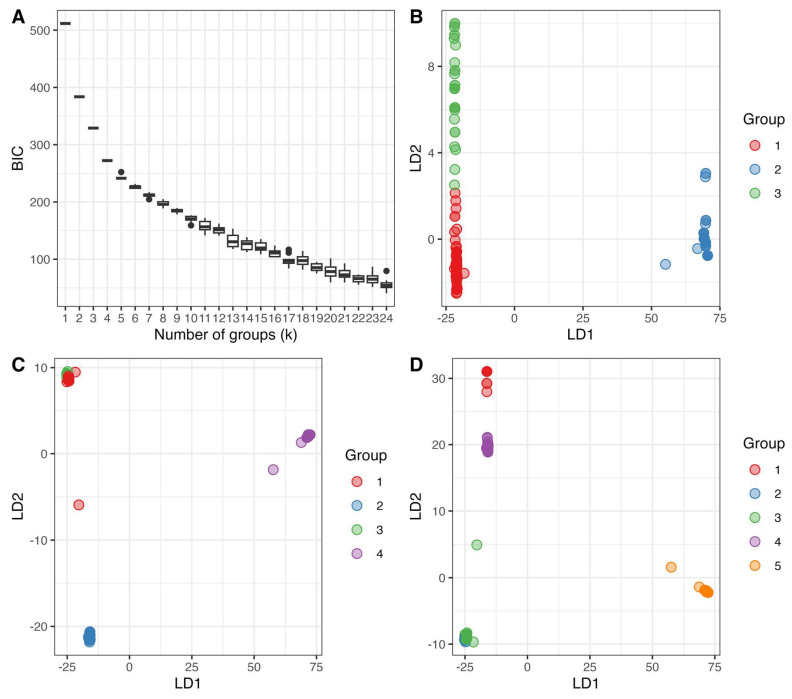
Bayesian Information Criterion (BIC) boxplot and Discriminant Analysis of Principal Components (DAPC) scatterplots based on mitogenome SNPs. (**A**) BIC boxplot for each putative grouping kernal (k) value for the country-wise clone corrected dataset. BIC values were obtained from 10 repetitions of the *find.clusters* function in *r*, which applies successive K-means on Principal Component Analysis (PCA) transformed data then measures for goodness of fit (BIC) of the model. (**B**) Scatter plot of the first two Discriminant Analysis of Principal Components (DAPC) loadings of the country wise clone corrected dataset when k = 3. Despite groups #1 and #3 clustering close to each other, there is little overlap between them. (**C**) Scatter plot of the first two DAPC loadings of the country wise clone corrected dataset when k = 4. Noticeable overlap between groups #1 and #3 can be seen in the top left corner of the plot. (**D**) Scatter plot of the first two DAPC loadings of the country wise clone corrected dataset when k = 5. Noticeable overlap between groups #2 and #3 can be seen in the bottom left corner of the plot.

The Bayesian Information Criterion (BIC) plot exhibited a sharp drop from a k value of one to two, followed by a less steep decline from two to three, and three to four (Figure 4A). Subsequent increases in k values showed minimal differences and less pronounced drops (Figure 4A). Plotting the first and second loadings for each grouping value between two and five revealed distinct clusters for a k value of three (Figure 4B). Increasing the k value from three to four resulted in cluster #3 being split into two clusters, #3 and #4 (Figure 4C,D). However, according to the NJ tree, with k = 4, one MLG, MLG 74, belonging to cluster #3, showed a closer phylogenetic relationship with cluster #4 MLGs than with the remaining cluster #3 MLGs. Similarly, two MLGs, MLGs 32 and 33, were categorized as cluster #4 despite a closer phylogenetic relationship with clusters #3 and #1.

### 3.4. Geographic Structure

To investigate the geographic structure of mitogenome SNPs and estimate the potential quantitative relationships between geographic distance and genetic distance among our samples, we conducted AMOVA at the continental and country levels and the Mantel analyses at the country level.

AMOVA was conducted using three different datasets: (1) the non-clone-corrected dataset including all countries with sample sizes greater than eight, (2) the same dataset after clone correction on a country-wise basis, and (3) the country-wise clone corrected dataset but included only countries with more than nine MLGs. The AMOVA test of the first dataset revealed a statistically significant genetic differentiation among countries, but the differentiation was statistically insignificant among continents. However, after clone correction, no statistically significant genetic differentiations were observed at either the country or the continental levels for both the second and third dataset. Taken together, the results suggested that localized clonal expansion of mitogenome MLGs were responsible for the observed statistically significant genetic differentiations in the total samples among countries.

**Figure 5:**
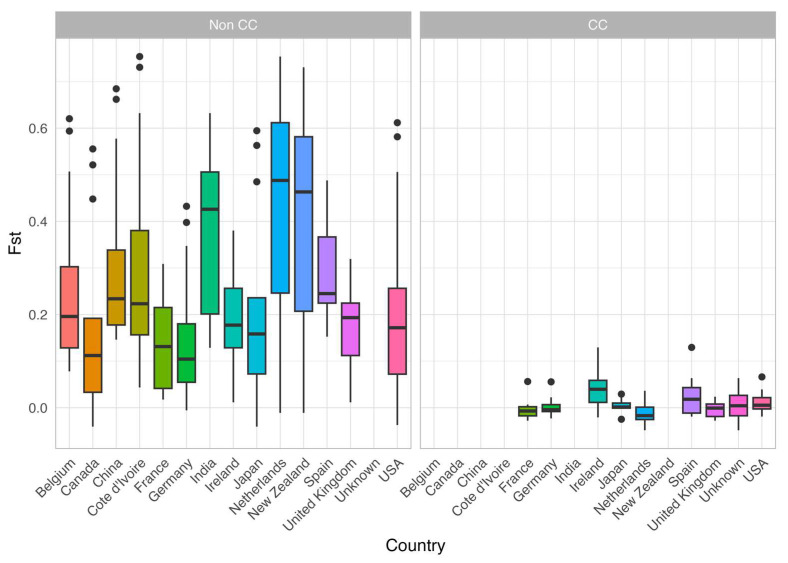
Pairwise Nei’s Fst values for countries with sample sizes exceeding 8 (**left**) or total MLGs exceeding 8 (**right**). The left panel was generated using the original sample data original sample data (non-CC), while the right consisted of country-wise clone corrected (CC) data (filtered for sample size post clone correction). The value for each pair of countries is represented twice in each figure, once towards each country in the pair. The thick horizontal line within each colored box is the median, each colored box includes the middle 50% of the data, the lines coming out the box/whiskers are the outer 50% of the data. The dots on top are observed Fst values that exceed 1.5x of the middle 50% of the data.

To further identify which pairs of national *A. fumigatus* samples were genetically differentiated, we obtained Fst values between pairs of national samples using both the non-clone-corrected data and the clone-corrected data. Similar to the analyses above, only countries with an initial sample size exceeding eight for the original dataset (i.e., dataset #1 above) and exceeding eight MLGs for clone-corrected data (i.e., dataset #3 above) were analyzed. The preliminary pairwise comparison revealed a considerable and diverse distribution of Fst values between countries (Figure 5). Among the 91 paired national samples in the non-clonal-corrected dataset, 51 pairs showed significant (*p* < 0.01, 999 permutations) differentiation. The two pairings that resulted in the highest Fst values both included Cote d’Ivoire. The two highest Fst values, 0.7538 and 0.7308, were observed between Cote d’Ivore and the Netherlands and New Zealand, respectively. However, after clone correction, there was a substantial reduction in variability among country pairs. In addition, six of the initial 14 countries assessed failed to meet our sample size criteria for comparison, and thus only eight countries were compared (Figure 5). As shown in Figure 5, clone-correction led to the reduction in pairwise Fst values across the board and none of the observed differences between countries were statistically significant in the clone-corrected samples. Consistent with the observations of frequent gene flows, the Mantel test revealed slightly positive but no statistically significant correlation between geographic distance and population genetic differentiation between pairs of national populations (the Mantel r statistic was 0.109 and a *p*-value of 0.271 based on 999 bootstrap iterations).

### 3.5. Recombination and Phylogenetic Incompatability

Three analyses were performed to investigate the possibility of recombination in the mitogenome in *A. fumigatus*. Here, only the global sample of 79 clone-corrected MLGs were included in the analyses. In the first analysis of multilocus index of association (I_A_) among the 39 SNP loci, our sample showed significant deviations from random mating and recombination, with the overall I_A_ and standardized rD values of 2.50 and 0.07, respectively. Both values were significantly higher than what would be expected in randomly recombining populations (*p* < 0.01). In the second test, phylogenetic compatibility analysis of the 39 SNP loci also demonstrated a significant departure from random mating. Approximately 80% of the loci pairs displayed phylogenetic compatibility, which is significantly higher than what would be observed under the hypothesis of random recombination (*p* < 0.01). In addition, out of the 741 SNP loci pairs, 73 were found to exhibit significant allelic associations, indicative of linkage disequilibrium (*p* < 0.01, Bonferroni correction). Taken together, these results indicated no evidence of random recombination in the mitogenome of the global *A. fumigatus* population.

**Figure 6:**
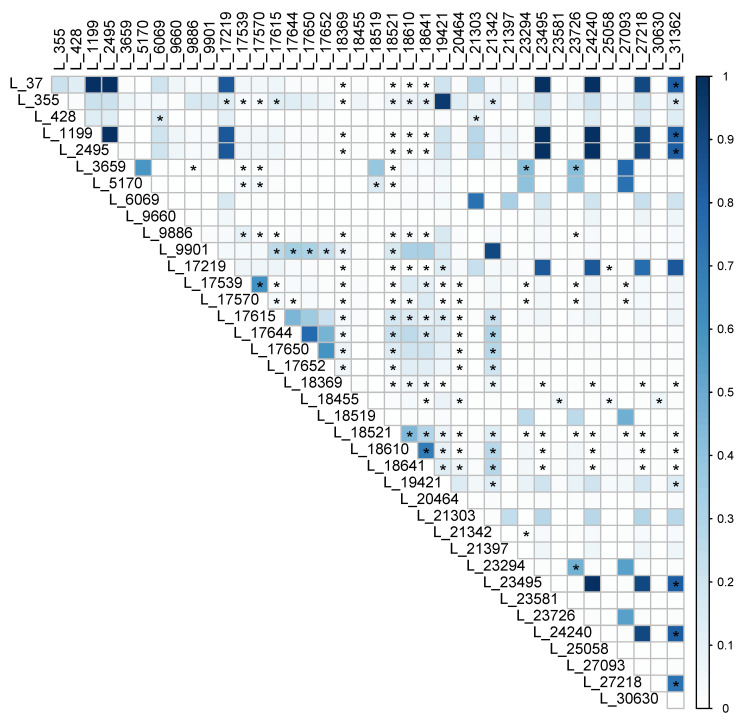
Allelic correlation/linkage disequilibrium and Four Gamete Test results. Color intensity represents r^2^ value, with higher color intensity indicating higher correlation. Pairs of loci marked with ‘*’ contained all four possible pairs of alleles, indicative of phylogenetic incompatibility and consistent with recombination.

However, signatures of recombination were found in the mitogenomes of this global sample. Specifically, 145 of the 741 pairs of SNP loci showed phylogenetic incompatibility (Figure 6), consistent with recombination between these SNP sites. In addition, upon comparison of the LD and four gamete test results per loci pair, the majority (86%) of the phylogenetically incompatible pairs failed to reject the null hypothesis of recombination (Figure 6). Taken together, our results suggest that while non-random mating and linkage disequilibrium dominate, there is unambiguous evidence for recombination in the mitogenomes of the global population of *A. fumigatus*. 

### 3.6. Reletive Mitogenome to Nuclear Genome Copy Number Ratio

**Figure 7:**
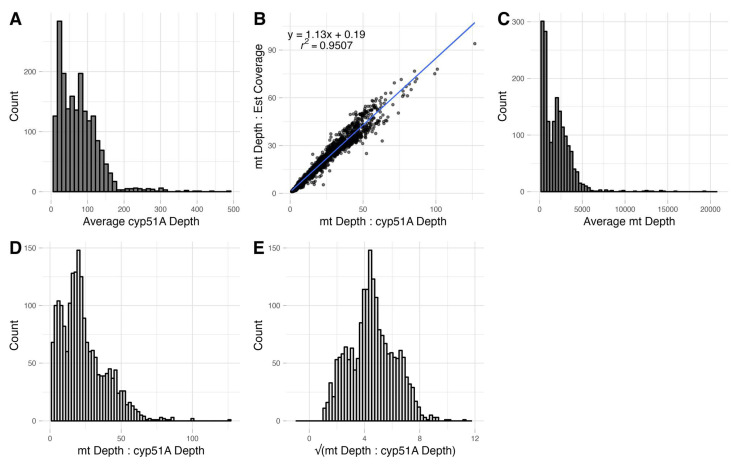
Distributions of sequence read depths and mitogenome to nuclear genome read depth ratios among 1939 isolates of *A. fumigatus*. (**A**) Right-skewed histogram of the average *cyp51A* read depth per isolate, n = 1939. (**B**) Relationship between mitogenome to *cyp51A* read depths ratio (x-axis) and the mitogenome read depth to estimated genome coverage ratio (y-axis). The values are strongly correlated with an r^2^ = 0.9507 (*p* < 0.01), n = 1929. (**C**) Right-skewed histogram of the average mt genome read depth per isolate, n = 1939. (**D**) Histogram of mt genome to *cyp51A* average read depths ratio displaying heavy right skew, n = 1929. (**E**) Histogram of the square root transformed values from D displaying a more normal distribution, n = 1929.

The average sequencing depth per nucleotide across the entire *cyp51A* gene varied widely among the 1939 isolates, from a few reads to over 400 reads (Figure 7A). As expected, the sequencing depths estimated based on *cyp51A* and the whole-genome coverage are highly correlated with each other (r^2^ = 0.95, *p* < 0.001; Figure 7B). Similarly, the average sequencing depth per nucleotide across the mitogenome varied widely among the 1939 isolates, from a few reads to over 15000 reads (Figure 7C). Both the *cyp51A* read depth and the mitogenome read depth data showed a heavy right skew, indicated by the high values of γ (shape parameter), 1.79 and 2.83, respectively (Figure 7A,C). Interestingly, the ratio between mitogenome sequencing depth and *cyp51A* sequencing depth also showed a very broad distribution, from less than 1 to over 100, with one ratio close to 600.

We checked the metadata and citations of strains with either very low or very high ratios. Our analyses identified that ten of the strains with either very high (>200) or very low (<1) ratios were either transcriptome data or synthetic metagenome data but were deposited as whole-genome sequence data. These ten strains were removed from subsequent analyses of mitogenome to nuclear genome ratios. Interestingly, though the raw ratios had a significantly skewed distribution (γ = 1.10), the square-rooted ratios had a normal distribution (γ = 0.18), with a mean ratio of 20.70 among the 1929 isolates (95% CI = (18.06, 23.34); Figure 7D).

To investigate the potential patterns of mitogenome to nuclear genome ratios among strains and among genetic and geographic populations, we compared the ratio patterns across both the DAPC grouping and among countries with sample size greater than 8. Kruskal–Wallis testing revealed statistically significant heterogeneity in ratio distribution within both the DAPC groups and the countries examined (*p* < 0.05, *p* < 0.01). Subsequent post hoc pairwise Wilcoxon rank sum testing uncovered significant (*p* < 0.05) differences between Group #3 and both Group #1 and Group #2. Specifically, the Group #3 strains had an overall higher mitogenome to nuclear genome ratio than those in Groups #1 and #2, 29 compared to 21 and 18, respectively. In contrast, no significant differences were observed between Groups #1 and #2. 

**Figure 8:**
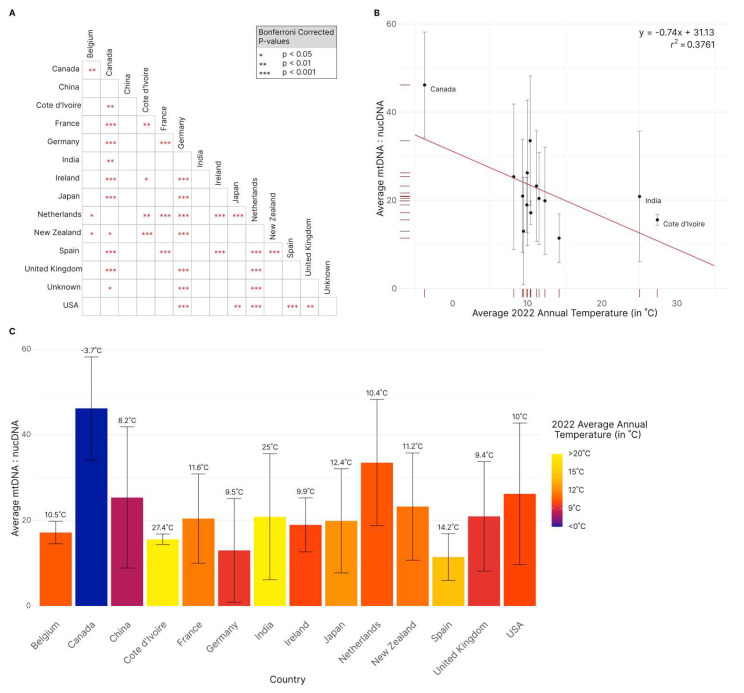
Comparisons between countries in the mitogenome to nuclear genome copy number ratios of strains of *Aspergillus fumigatus* and its relationship to average temperature in 2022. (**A**). Pairwise Wilcoxon Rank Sum Tests between countries in their mitogenome to nuclear genome copy number ratios. Significance is denoted by Bonferroni corrected *p*-values where (*) indicates 95%, (**) indicates 99%, and (***) indicates 99.9% confidence. (**B**). Scatterplot of countries’ average ratio (± 1 sd) of mitochondria DNA to nuclear DNA against average annual temperature for 2022 (in °C). An overall negative correlation between ratio and temperature (y = −0.74x + 31.13, *p* < 0.05, *r*^2^ = 0.3761) was found. Countries at the two ends of temperature distributions are labeled. (**C**). Average ratio (± 1 sd) of mitochondrial DNA to nuclear DNA per country. Average 2022 annual temperature in degrees Celsius, is indicated by both color and above each bar. Notably, Canada is 11.9 °C lower than the next coldest country and the only country with an average annual temperature below 0 °C. For all figures, only countries with sample size greater than eight isolates were included.

Among the 14 compared countries (each country with sample size greater than 8 isolates), 38 pairs (out of 91 total pairs) showed statistically significant (*p* < 0.05, Bonferroni corrected) differences in their mitogenome to nuclear genome ratios (Figure 8A). Canada had the highest average ratio of 46.2 mitochondrial to nuclear genome depth ratios, more than double the overall average of 20.7 (Figure 8). Conversely, Spain had the lowest average ratio, 11.4, just over half of the overall average (Figure 8). Canada had a significantly higher ratio than 11 of the 13 compared countries. In contrast, Spain had a significantly lower ratio than 6 of the 13 compared countries. Interestingly, China stood out as the only country that did not display any significant differences when compared to other 13 countries, primarily due to the large variations among strains within China. Furthermore, China and Canada were not significantly different, but Canada and New Zealand were (at a 0.05 significance level) despite New Zealand having a higher average ratio than China (22.0 and 20.0, respectively) (Figure 8). Interestingly, there is evidence of a statistically significant negative correlation between the mitochondrial to nuclear genome ratio and climate. Comparing the samples from the 14 countries to the average annual temperature for 2021 resulted in a Pearson correlation coefficient of −0.61 (*p* < 0.05) (Figure 8B).

## 4. Discussion

In this study, we conducted a broad analysis of mitogenome SNPs in a global collection of 1939 isolates of *A. fumigatus*, a critical priority human fungal pathogen. Our analyses identified 39 SNPs and 79 multi-locus genotypes among the analyzed isolates. In addition, we found a range of mitogenome to nuclear genome copy number ratios, from about 11 to over 100, with a mean of ~20. Our examination of concatenated SNP sequences separated the strains into at least three genetic groups. However, though multiple genotypes are often overrepresented in certain geographic regions, there was limited geography-based clustering at either the country or the continent level for the 79 mitochondrial genotypes. Interestingly, evidence for mitogenome recombination was found between several pairs of SNP loci. Below, we discuss the relevance and potential implications of our results.

### 4.1. Mitogenome SNPs

In this study, 39 SNPs were identified among the 1939 isolates from around the world. Among the 24 SNPs in protein-coding genes, 3 were synonymous while 21 were nonsynonymous. This high ratio of nonsynonymous to synonymous SNPs suggested potential positive selection might have acted on the mitogenome during the evolution of A. fumigatus. However, all nonsynonymous mutations were predicted to have low to moderate effects on protein structure and functions. Indeed, the two most frequently and most broadly distributed mitogenome MLGs, MLG11 and MLG79, were found to differ by six nonsynonymous mutations. At present, the functional impacts of these mutations are not known. Additional functional comparisons are needed in order to determine their potential roles in nature. Despite the uncertainty of functional importance of the observed mitogenome SNPs, mitogenome sequence variations provided valuable information for understanding natural populations of A. fumigatus.

### 4.2. Geographic Structuring

The analyzed global population included 1939 *A. fumigatus* isolates representing 22 countries in 6 continents, including two strains from the International Space Station. Among the 79 MLGs from these 1939 isolates, 19 were each distributed in at least three countries, indicative of continental and global clonal expansion. Indeed, all the sequenced samples from Africa, Oceana, South America, and the International Space Station shared MLGs with samples from other geographic regions and none had private mitochondrial MLGs. Interestingly, the two samples from the International Space Station were genetically divergent from each other, similar to the extensive differences found in their nuclear genomes [3]. On the other hand, 40 of the 79 MLGs were found only in one country each, and AMOVA analysis of the total samples revealed statistically significant genetic differentiations among national samples. However, clone-corrected samples revealed limited genetic differentiations among national samples. Together, the results suggested that localized clonal expansion of certain MLGs played a major role in the observed genetic differentiations among countries in the total sample. For instance, MLGs 11 and 79 were the most abundant, representing 420 isolates from 18 countries and 418 isolates from 11 countries, respectively (Figure 1). However, over half and one third of MLG 11 and MLG 79 isolates were found in the Netherlands and the USA, respectively. Localized clonal expansion can significantly bias allele frequencies, contributing to the observed genetic difference among geographic populations [50].

Our phylogenetic and DAPC analyses showed our samples can be optimally clustered into three major clades. This finding differed from previous studies conducted by Pringle et al. [51], Klassen et al. [52], and Ashu et al. [53] who found two, five, and eight clades/genetic clusters, respectively, in their global samples. These three studies used different markers as ours: they all relied on nuclear genomic data, based on either multilocus sequence typing or microsatellite markers. Interestingly, while both Pringle et al. [51] and Klassen et al. [52] conclude no significant correlation between clades and geography, Ashu et al. [53] found limited but statistically significant geographic contributions to genetic variation with a statistically significant geographic contributions in the non-clone corrected AMOVA, but a non-significant Mantel test. The difference in population structure among the studies likely reflect the different samples used and the difference in inheritance patterns between nuclear and mitochondrial genomes, especially where mitochondria largely inherit in a non-Mendelian pattern during sexual mating in filamentous ascomycetes [17]. 

We note that despite the large sample size (1939 isolates) and broad geographic representation (22 countries from across six continents), the analyzed samples were still limited in their geographic distributions. Specifically, the 22 countries represented only about 12% of the total countries in the world. A previous study by Ashu et al. [53] revealed the Cameroonian population of *A. fumigatus* to be genetically highly distinct from other national populations. It is likely that the mitogenomes from the Cameroonian populations will be genetically distinct as well. However, there is currently no genome sequence information from Cameroonian isolates of *A. fumigatus*. In addition, among the 22 sampled countries, the distributions of isolates were also highly skewed. Specifically, over half of the isolates (1036) originated from western Europe, and an additional 30% (563) were from the United States. Furthermore, we were unable to find the geographic origins for 114 of the isolates. Together, we believe that better data submission and including more strains from under-sampled regions and strains from not-yet sampled regions will reveal additional novel mitogenome diversity in this species.

### 4.3. Mitogenome Recombination

We observed signatures of recombination in the mitogenome of this global population of *A. fumigatus*. In *A. fumigatus*, the mitochondrial inheritance has not been critically examined in sexual crosses in the lab. However, sexual mating through hyphal fusion between strains of different mating types should result in significant cytoplasmic mixing, including the potential for mitochondrial fusion and mitogenome recombination. The observation of cleistothecia development along junction zones between mating partners is consistent with hyphal fusion being a dominant mode of mating in *A. fumigatus* [54]. Our observed mitogenome recombination in natural populations of *A. fumigatus* contrasts with those observed in the majority of eukaryotic organisms, where mitogenomes are inherited uniparentally in sexual crosses without recombination [17]. However, a growing number of fungal species have shown evidence of mitogenome recombination in their natural populations [17,55,56]. Interestingly, studies on other members of the *Aspergillus* genus have provided potential evidence of intra- and inter-species mitochondrial recombination. For instance, investigations on *Aspergillus nidulans* using genetic crosses, gel electrophoresis, Southern hybridization, and electron microscopy suggested genetic exchange between mitogenomes [57,58]. Similarly, novel mitogenomes were observed in the progeny of a sexual cross in *Aspergillus niger*, a related opportunistic human pathogen, caused by the homing of mobile mitochondrial group I introns [59]. In both cases, restriction fragment length differences were observed between parental and progeny mitogenomes, consistent with horizontal transfer of mobile introns. However, in our *A. fumigatus* natural populations, evidence for recombination was observed between SNPs located across the mitogenome.

### 4.4. Relative Copy Number Ratios of Mitogenome to Nuclear Genome

Aside from mitogenome SNPs, we also investigated the relative copy number differences between mitochondrial and nuclear genomes among the 1939 strains of *A. fumigatus*. Wide variations of the ratio, from about 11 to over 100, were found among strains and among geographic populations. Based on the redundancy in their assembled sequence, a previous study estimated a clinical isolate of *A. fumigatus* Af293 having a mitochondrial to nuclear DNA ratio of ~12:1 [60]. In a more recent study based on a real-time PCR of single copy genes in the nuclear and mitochondrial genomes, Neubauer et al. [61] estimated that the mitochondrial to nuclear genome ratio in a wild-type strain AfS35 was about 60(±15):1. At present, the underlying mechanisms for such variations are unknown. Based on the literature, all the 1929 genome-sequenced strains were grown in favorable laboratory conditions before their mycelia were harvested and DNA extracted for sequencing. While some variations may exist in growth conditions among studies in preparing their strains for DNA extraction and genome sequencing, the observed differences among geographic populations suggest that the identified range of ratios might reflect their adaptations to the natural environments from which the strains originally resided. Specifically, we hypothesize that the relatively high mitochondrial to nuclear genome ratio for Canadian isolates may reflect the low temperature these isolates were adapted to and the need to have a high number of mitochondrial genes to generate more ATP to fulfill their metabolic needs in such environments. Controlled experimental evolution under different temperatures are needed to test this hypothesis. Furthermore, due to potential differences in their capacity to generate ATP among strains with different mitochondrial to nuclear genome ratios, we speculate that such strains may exhibit different properties such as antifungal drug resistance and pathogenesis. Targeted experimental studies are needed to determine the biological significance of the observed variations in mitochondrial to nuclear genome ratios among strains.

## 5. Conclusions and Perspectives

Our study provides novel insights into mitogenome variations in a large global collection isolates of *A. fumigatus*, a critical human fungal pathogen. Our analyses revealed evidence for both global and local clonal expansions of mitogenomes. The over-representation of relatively few mitochondrial genotypes in the global sample suggested potential selective advantages of these mitochondrial MLGs and/or their associated nuclear genomes in both natural environments and patients. Due to their biased clinical prevalence, whenever possible, the overrepresented mitogenome MLGs should be used for virulence and drug development studies. The identified mitogenome SNPs and MLGs could be used as molecular markers to track future epidemiological patterns of *A. fumigatus* at both local and global scales. In addition, evidence of mitogenome recombination was found among several mtSNP loci, indicating a departure from typical uniparental mitochondrial inheritance pattern in eukaryotes. Similarly, the investigation of relative copy number differences between mitochondria and nuclear genomes revealed a wide range of ratios. At present, the potential biological significance of the observed variations in mitogenome to nuclear genome ratios is not known. However, it’s tempting to speculate that the variable ratios likely reflect the dynamic nature of mitochondrial genome replication and maintenance, which are regulated by various environmental and metabolic cues. Our study underscores the need for further investigation into the functional implications of the observed mitogenome variations in *A. fumigatus*. Collectively, these findings help lay the foundation for future studies into the genetic mechanisms underlying the diversity and evolution of this critical fungal pathogen.

## Figures and Tables

**Table 1:** Summary geographic distribution of the *A. fumigatus* isolates analyzed in this study. Two geographic levels are shown: the country level and the continental level. For each region, total samples (N), percentage of total dataset (%), and number of both private and total mitogenome multi-locus genotypes (MLGs) are reported.

Region	*N*	%	Private MLG	Total MLG	Region	*N*	%	Private MLG	Total MLG
Africa	9	0.46%	0	2	Europe	1036	53.43%	20	56
Cote d’Ivoire	9	0.46%	0	2	Austria	2	0.10%	0	1
Asia	181	9.33%	4	24	Belgium	10	0.52%	0	5
China	10	0.52%	0	4	France	161	8.30%	3	26
India	12	0.62%	1	3	Germany	262	13.51%	7	30
Iran	2	0.10%	2	2	Ireland	72	3.71%	3	17
Japan	155	7.99%	1	18	Netherlands	282	14.54%	1	17
Singapore	1	0.05%	0	1	Portugal	8	0.41%	0	5
Thailand	1	0.05%	0	1	Spain	28	1.44%	4	14
Oceania	24	1.24%	0	5	Sweden	1	0.05%	0	1
New Zealand	24	1.24%	0	5	United Kingdom	210	10.83%	2	25
North America	573	29.55%	13	39	South America	2	0.10%	0	2
Canada	10	0.52%	1	6	Brazil	1	0.05%	0	1
USA	563	29.33%	12	38	Peru	1	0.05%	0	1
Space	2	0.10%	0	2	Unknown	112	5.78%	3	18
ISS	2	0.10%	0	2	Grand Total	1939	100.00%	40	79

## Data Availability

All data described in this study are presented in the manuscript and public available.

## Supplementary Materials

The following supporting information can be downloaded at: https://www.mdpi.com/article/10.3390/jof9100995/s1, Table S1: Information about the 1939 isolates analyzed in this study. Table S2: Annotation of mitogenome SNPs observed among 1939 isolates of *A. fumigatus*.

## References

[1] Xu J. (2022). Assessing Global Fungal Threats to Humans. mLife.

[2] Latgé J.-P., Chamilos G. (2019). *Aspergillus fumigatus* and Aspergillosis in 2019. Clin. Microbiol. Rev..

[3] Knox B.P., Blachowicz A., Palmer J.M., Romsdahl J., Huttenlocher A., Wang C.C.C., Keller N.P., Venkateswaran K. (2016). Characterization of *Aspergillus fumigatus* Isolates from Air and Surfaces of the International Space Station. mSphere.

[4] Paulussen C., Hallsworth J.E., Álvarez-Pérez S., Nierman W.C., Hamill P.G., Blain D., Rediers H., Lievens B. (2017). Ecology of Aspergillosis: Insights into the Pathogenic Potency of *Aspergillus fumigatus* and Some Other *Aspergillus* Species. Microb. Biotechnol..

[5] Nywening A.V., Rybak J.M., Rogers P.D., Fortwendel J.R. (2020). Mechanisms of Triazole Resistance in *Aspergillus fumigatus*. Environ. Microbiol..

[6] Dagenais T.R.T., Keller N.P. (2009). Pathogenesis of *Aspergillus fumigatus* in Invasive Aspergillosis. Clin. Microbiol. Rev..

[7] World Health Organization (2022). WHO Fungal Priority Pathogens List to Guide Research, Development and Public Health Action.

[8] Barber A.E., Sae-Ong T., Kang K., Seelbinder B., Li J., Walther G., Panagiotou G., Kurzai O. (2021). *Aspergillus fumigatus* Pan-Genome Analysis Identifies Genetic Variants Associated with Human Infection. Nat. Microbiol..

[9] Szalewski D.A., Hinrichs V.S., Zinniel D.K., Barletta R.G. (2018). The Pathogenicity of *Aspergillus fumigatus*, Drug Resistance, and Nanoparticle Delivery. Can. J. Microbiol..

[10] Black B., Lee C., Horianopoulos L.C., Jung W.H., Kronstad J.W. (2021). Respiring to Infect: Emerging Links between Mitochondria, the Electron Transport Chain, and Fungal Pathogenesis. PLOS Pathog..

[11] Grahl N., Dinamarco T.M., Willger S.D., Goldman G.H., Cramer R.A. (2012). *Aspergillus fumigatus* Mitochondrial Electron Transport Chain Mediates Oxidative Stress Homeostasis, Hypoxia Responses and Fungal Pathogenesis. Mol. Microbiol..

[12] Ma H., Hagen F., Stekel D.J., Johnston S.A., Sionov E., Falk R., Polacheck I., Boekhout T., May R.C. (2009). The Fatal Fungal Outbreak on Vancouver Island Is Characterized by Enhanced Intracellular Parasitism Driven by Mitochondrial Regulation. Proc. Natl. Acad. Sci. USA.

[13] Magnani T., Soriani F.M., Martins V. (2008). de P.; Policarpo, A.C.d.F.; Sorgi, C.A.; Faccioli, L.H.; Curti, C.; Uyemura, S.A. Silencing of Mitochondrial Alternative Oxidase Gene of *Aspergillus fumigatus* Enhances Reactive Oxygen Species Production and Killing of the Fungus by Macrophages. J. Bioenerg. Biomembr..

[14] Misslinger M., Lechner B.E., Bacher K., Haas H. (2018). Iron-Sensing Is Governed by Mitochondrial, Not by Cytosolic Iron–Sulfur Cluster Biogenesis in *Aspergillus fumigatus*. Metallomics.

[15] Song J., Zhou J., Zhang L., Li R. (2020). Mitochondria-Mediated Azole Drug Resistance and Fungal Pathogenicity: Opportunities for Therapeutic Development. Microorganisms.

[16] Basse C.W. (2010). Mitochondrial Inheritance in Fungi. Curr. Opin. Microbiol..

[17] Sandor S., Zhang Y., Xu J. (2018). Fungal Mitochondrial Genomes and Genetic Polymorphisms. Appl. Microbiol. Biotechnol..

[18] Yan Z., Xu J. (2005). Fungal mitochondrial inheritance and evolution. Evolutionary Genetics of Fungi.

[19] Joardar V., Abrams N.F., Hostetler J., Paukstelis P.J., Pakala S., Pakala S.B., Zafar N., Abolude O.O., Payne G., Andrianopoulos A. (2012). Sequencing of Mitochondrial Genomes of Nine *Aspergillus* and *Penicillium* Species Identifies Mobile Introns and Accessory Genes as Main Sources of Genome Size Variability. BMC Genom..

[20] Song S.-N., Tang P., Wei S.-J., Chen X.-X. (2016). Comparative and Phylogenetic Analysis of the Mitochondrial Genomes in Basal Hymenopterans. Sci. Rep..

[21] Lewin R. (1987). The Unmasking of Mitochondrial Eve. Science.

[22] Wang Y., Xu J. (2020). Mitochondrial Genome Polymorphisms in the Human Pathogenic Fungus *Cryptococcus neoformans*. Front. Microbiol..

[23] Hebert P.D.N., Cywinska A., Ball S.L., deWaard J.R. (2003). Biological Identifications through DNA Barcodes. Proc. Biol. Sci..

[24] Xu J. (2016). Fungal DNA Barcoding. Genome.

[25] Kozlowski M., Stepien P.P. (1982). Restriction Enzyme Analysis of Mitochondrial DNA of Members of the Genus *Aspergillus* as an Aid in Taxonomy. Microbiology.

[26] Wang L., Yokoyama K., Miyaji M., Nishimura K. (1998). The Identification and Phylogenetic Relationship of Pathogenic Species of Aspergillus Based on the Mitochondrial Cytochrome b Gene. Med. Mycol..

[27] Wang L., Yokoyama K., Miyaji M., Nishimura K. (2000). Mitochondrial Cytochrome b Gene Analysis of *Aspergillus fumigatus* and Related Species. J. Clin. Microbiol..

[28] Álvarez-Iglesias V., Mosquera-Miguel A., Cerezo M., Quintáns B., Zarrabeitia M.T., Cuscó I., Lareu M.V., García Ó., Pérez-Jurado L., Carracedo Á. (2009). New Population and Phylogenetic Features of the Internal Variation within Mitochondrial DNA Macro-Haplogroup R0. PLoS ONE.

[29] Chung H. (2013). Phylogenetic Analysis and Characterization of Mitochondrial DNA for Korean Native Cattle. Open J. Genet..

[30] Sabir J.S.M., Arasappan D., Bahieldin A., Abo-Aba S., Bafeel S., Zari T.A., Edris S., Shokry A.M., Gadalla N.O., Ramadan A.M. (2014). Whole Mitochondrial and Plastid Genome SNP Analysis of Nine Date Palm Cultivars Reveals Plastid Heteroplasmy and Close Phylogenetic Relationships among Cultivars. PLoS ONE.

[31] Zhu Q., Gao P., Liu S., Amanullah S., Luan F. (2016). Comparative Analysis of Single Nucleotide Polymorphisms in the Nuclear, Chloroplast, and Mitochondrial Genomes in Identification of Phylogenetic Association among Seven Melon (*Cucumis melo* L.) Cultivars. Breed. Sci..

[32] Barber A.E., Riedel J., Sae-Ong T., Kang K., Brabetz W., Panagiotou G., Deising H.B., Kurzai O. (2020). Effects of Agricultural Fungicide Use on *Aspergillus fumigatus* Abundance, Antifungal Susceptibility, and Population Structure. mBio.

[33] Hagiwara D., Takahashi H., Watanabe A., Takahashi-Nakaguchi A., Kawamoto S., Kamei K., Gonoi T. (2014). Whole-Genome Comparison of *Aspergillus fumigatus* Strains Serially Isolated from Patients with Aspergillosis. J. Clin. Microbiol..

[34] Lofgren L.A., Ross B.S., Cramer R.A., Stajich J.E. (2022). The Pan-Genome of Aspergillus Fumigatus Provides a High-Resolution View of Its Population Structure Revealing High Levels of Lineage-Specific Diversity Driven by Recombination. PLOS Biol..

[35] Rhodes J., Abdolrasouli A., Dunne K., Sewell T.R., Zhang Y., Ballard E., Brackin A.P., van Rhijn N., Chown H., Tsitsopoulou A. (2022). Population Genomics Confirms Acquisition of Drug-Resistant *Aspergillus fumigatus* Infection by Humans from the Environment. Nat. Microbiol..

[36] Winter D.J., Weir B.S., Glare T., Rhodes J., Perrott J., Fisher M.C., Stajich J.E., Digby A., Dearden P.K., Cox M.P. (2022). A Single Fungal Strain Was the Unexpected Cause of a Mass Aspergillosis Outbreak in the World’s Largest and Only Flightless Parrot. iScience.

[37] Chen S., Zhou Y., Chen Y., Gu J. (2018). Fastp: An Ultra-Fast All-in-One FASTQ Preprocessor. Bioinformatics.

[38] Li H. (2013). Aligning Sequence Reads, Clone Sequences and Assembly Contigs with BWA-MEM. arXiv.

[39] Danecek P., Bonfield J.K., Liddle J., Marshall J., Ohan V., Pollard M.O., Whitwham A., Keane T., McCarthy S.A., Davies R.M. (2021). Twelve Years of SAMtools and BCFtools. GigaScience.

[40] McKenna A., Hanna M., Banks E., Sivachenko A., Cibulskis K., Kernytsky A., Garimella K., Altshuler D., Gabriel S., Daly M. (2010). The Genome Analysis Toolkit: A MapReduce Framework for Analyzing Next-Generation DNA Sequencing Data. Genome Res..

[41] Ortiz E.M. (2019). Vcf2phylip v2.0: Convert a VCF Matrix into Several Matrix Formats for Phylogenetic Analysis. https://zenodo.org/record/2540861.

[42] Gkanogiannis A. fastreeR: Phylogenetic, Distance and Other Calculations on VCF and Fasta Files 2023. https://bioconductor.org/packages/release/bioc/manuals/fastreeR/man/fastreeR.pdf.

[43] Paradis E., Schliep K. (2019). Ape 5.0: An Environment for Modern Phylogenetics and Evolutionary Analyses in R. Bioinformatics.

[44] Letunic I., Bork P. (2021). Interactive Tree of Life (iTOL) v5: An Online Tool for Phylogenetic Tree Display and Annotation. Nucleic Acids Res..

[45] Kamvar Z.N., Tabima J.F., Grünwald N.J. (2014). *Poppr*: An R Package for Genetic Analysis of Populations with Clonal, Partially Clonal, and/or Sexual Reproduction. PeerJ.

[46] Jombart T., Devillard S., Balloux F. (2010). Discriminant Analysis of Principal Components: A New Method for the Analysis of Genetically Structured Populations. BMC Genet..

[47] Brown A.H.D., Feldman M.W., Nevo E. (1980). Multilocus structure of natural populations of *Hordeum spontaneum*. Genetics.

[48] Smith J.M., Smith N.H., O’Rourke M., Spratt B.G. (1993). How Clonal Are Bacteria?. Proc. Natl. Acad. Sci. USA.

[49] Wei T., Simko V. (2021). R Package “Corrplot”: Visualization of a Correlation Matrix. https://github.com/taiyun/corrplot.

[50] Xu J. (2006). Fundamentals of Fungal Molecular Population Genetic Analyses. Curr. Issues Mol. Biol..

[51] Pringle A., Baker D.M., Platt J.L., Wares J.P., Latgé J.P., Taylor J.W. (2005). Cryptic Speciation in the Cosmopolitan and Clonal Human Pathogenic Fungus *Aspergillus fumigatus*. Evolution.

[52] Klassen C.H.W., Gibbons J.G., Fedorova N.D., Meis J.F., Rokas A. (2012). Evidence for Genetic Differentiation and Variable Recombination Rates among Dutch Populations of the Opportunistic Human Pathogen *Aspergillus fumigatus*. Mol. Ecol..

[53] Ashu E.E., Hagen F., Chowdhary A., Meis J.F., Xu J. (2017). Global Population Genetic Analysis of *Aspergillus fumigatus*. mSphere.

[54] Verma S., Shakya V.P.S., Idnurm A. (2018). Exploring and Exploiting the Connection between Mitochondria and the Virulence of Human Pathogenic Fungi. Virulence.

[55] Korfanty G., Stanley K., Lammers K., Fan Y.Y., Xu J. (2021). Variations in Sexual Fitness among Natural Strains of the Opportunistic Human Fungal Pathogen *Aspergillus fumigatus*. Infect. Genet. Evol..

[56] Wang Y., Xu J. (2022). Population Genomic Analyses Reveal Evidence for Limited Recombination in the Superbug *Candida auris* in Nature. Comput. Struct. Biotechnol. J..

[57] Earl A.J., Turner G., Croft J.H., Dales R.B.G., Lazarus C.M., Lünsdorf H., Küntzel H. (1981). High Frequency Transfer of Species-Specific Mitochondrial DNA Sequences between Members of the Aspergillaceae. Curr. Genet..

[58] Rowlands R.T., Turner G. (1975). Three-Marker Extranuclear Mitochondrial Crosses in *Aspergillus nidulans*. Molec. Gen. Genet..

[59] Hamari Z., Tóth B., Beer Z., Gácser A., Kucsera J., Pfeiffer I., Juhász Á., Kevei F. (2003). Interpretation of Intraspecific Variability in mtDNAs of *Aspergillus niger* Strains and Rearrangement of Their mtDNAs Following Mitochondrial Transmissions. FEMS Microbiol. Lett..

[60] Nierman W.C., Pain A., Anderson M.J., Wortman J.R., Kim H.S., Arroyo J., Berriman M., Abe K., Archer D.B., Bermejo C. (2005). Genomic Sequence of the Pathogenic and Allergenic Filamentous Fungus *Aspergillus fumigatus*. Nature.

[61] Neubauer M., Zhu Z., Penka M., Helmschrott C., Wagener N., Wagener J. (2015). Mitochondrial Dynamics in the Pathogenic Mold *Aspergillus fumigatus*: Therapeutic and Evolutionary Implications. Mol. Microbiol..

